# Assessment of epicutaneous testing of a monovalent Influenza A (H1N1) 2009 vaccine in egg allergic patients

**DOI:** 10.1186/1710-1492-7-3

**Published:** 2011-02-11

**Authors:** Tracy Pitt, Chrystyna Kalicinsky, Richard Warrington, Nestor Cisneros

**Affiliations:** 1 Section of Allergy & Clinical Immunology, Health Sciences Centre, Winnipeg, Manitoba, Canada

## Abstract

**Background:**

H1N1 is responsible for the first influenza pandemic in 41 years. In the fall of 2009, an H1N1 vaccine became available in Canada with the hopes of reducing the overall effect of the pandemic. The purpose of this study was to assess the safety of administering 2 different doses of a monovalent split virus 2009 H1N1 vaccine in egg allergic patients.

**Methods:**

Patients were skin tested to the H1N1 vaccine in the outpatient paediatric and adult allergy and immunology clinics of the Health Sciences Centre and Children's Hospital of Winnipeg, Manitoba Canada. Individuals <9 years of age were administered 1.88 μg's of hem-agglutinin antigen per 0.25 ml dose and individuals ≥9 years were administered 3.75 μg's of hemagglutinin antigen per 0.5 ml dose. Upon determination of a negative skin test, the vaccine was administered with a 30 minute observation period.

**Results:**

A total of 61 patients with egg allergy (history of an allergic reaction to egg with either positive skin test &/or specific IgE to egg >0.35 Ku/L) were referred to our allergy clinics for skin testing to the H1N1 vaccine. 2 patients were excluded, one did not have a skin prick test to the H1N1 vaccine (only vaccine administration) and the other passed an egg challenge during the study period. Ages ranged from 1 to 27 years (mean 5.6 years). There were 41(69.5%) males and 18(30.5%) females. All but one patient with a history of egg allergy, positive skin test to egg and/or elevated specific IgE level to egg had negative skin tests to the H1N1 vaccine. The 58 patients with negative skin testing to the H1N1 vaccine were administered the vaccine and observed for 30 minutes post vaccination with no adverse results. The patient with the positive skin test to the H1N1 vaccine was also administered the vaccine intramuscularly with no adverse results.

**Conclusions:**

Despite concern regarding possible anaphylaxis to the H1N1 vaccine in egg allergic patients, in our case series 1/59(1.7%) patients with sensitization to egg were also sensitized to the H1N1 vaccine. Administration of the H1N1 vaccine in egg allergic patients with negative H1N1 skin tests and observation is safe. Administering the vaccine in a 1 or 2 dose protocol without skin testing is a reasonable alternative as per the CSACI guidelines.

## Background

The current swine-origin influenza A (H1N1) strain (S-OIV), also known as the swine flu, was responsible for numerous emergency room visits, hospital admissions, complications and deaths worldwide in 2009. A recent Canadian review of 58 children admitted to the Hospital for Sick Children with H1N1 influenza, found that 29% of these children had radiographic changes compatible with pneumonia. In addition, these children were significantly more likely to have a comorbid diagnosis of asthma than children with seasonal influenza [1]. The burden of illness as demonstrated by hospital admissions was especially evident amongst both pediatric patients and the elderly. A review of laboratory-confirmed cases in Ontario found the highest risk of hospitalization was among infants less than 1 year of age and in the elderly greater than 65 years of age [2]. The province of Manitoba was especially affected in 2009. One series reports 894 confirmed cases of H1N1 in Manitoba from April 2^nd ^to September 25^th ^2009, of whom 23% were admitted to hospital and 6% to the intensive care unit [3].

The rapid global spread of this virus prompted the World Health Organization to declare an H1N1 pandemic in June of 2009. In the fall of 2009, a vaccine was introduced in hopes of reducing the burden of this virus. Patients with IgE mediated allergy to egg have in the past been advised to avoid the influenza vaccine due to the egg content of the vaccine. Influenza vaccines are developed by inoculating embryonated chicken eggs with virus and thus contain ovalbumin. A recent review of ovalbumin content in H1N1 vaccines found a range of actual ovalbumin content of 0.064-1.411 mcg/ml [4]. The purpose of this study was to assess the safety of administering 2 different doses of a monovalent split virus 2009 H1N1 vaccine in egg allergic patients.

## Methods

Patients with a history of probable egg allergy and candidates for receiving the H1N1 vaccine were referred to the pediatric and adult allergy and immunology clinics for administration of the H1N1 vaccine. A patient was labeled as egg allergic if they had a convincing clinical history of an IgE mediated reaction to egg ingestion (including symptoms of urticaria, angioedema, cough, other breathing difficulties, wheeze, pruritus, flushing, rhinoconjunctivitis, throat tightness, gastrointestinal complaints, cyanosis, and circulatory collapse) within 2 hours. They either had positive epicutaneous testing to egg white allergen and/or were immunocap positive to egg as part of confirming the clinical diagnosis.

The Canadian Society of Allergy and Clinical Immunology (CSACI) recommends administration of the H1N1 vaccine based on 2 risk categories of patient with egg allergy [5]. The first category are lower risk patients(with mild gastrointestinal or mild local skin reaction, tolerating ingestion of small amounts of egg, or positive skin/specific IgE test to egg without knowingly exposed to egg) or higher risk (previous respiratory or cardiovascular reaction, generalized hives or those with poorly controlled asthma). For lower risk patients the vaccine is recommended to be administered with a 60 minute observation period. For patients at higher risk, or if the risk is unknown, an initial test dose with 10% of the total dose followed by 30 minutes of observation. If there is no reaction after 30 minutes, the remaining 90% of the vaccine can be given with observation for 60 minutes. Children who tolerate the split dose and who require a second dose (first time receiving influenza vaccine) can receive the next dose in one injection. For all of the patients in this study, it was decided that if a skin test was found to be negative, the entire dose of the vaccine would be given with a 30 minute observation period.

Patients were skin tested to the H1N1 vaccine in the outpatient pediatric and adult allergy and immunology clinics of the Health Sciences Centre and Children's Hospital of Winnipeg, Manitoba, Canada. Upon determination of a negative skin test, the vaccine was administered with a 30 minute observation period. Children under 9 years of age were administered 1.88 μg's of hem-agglutinin antigen per 0.25 ml dose and individuals ≥ 9 years were administered 3.75 μg's of hemagglutinin antigen per 0.5 ml dose intramuscularly based on the above protocol. Control subjects consisted of patients with no history of egg allergy, who were referred to the allergy and immunology clinics due to various reasons including a history of a previous reaction to the influenza vaccine.

Epicutaneous testing was performed via the prick lanceter method using egg white extract (ALK Abelló), AREPANRIX H1N1 AS03 Adjuvanted H1N1 vaccine (GlaxoSmithKline), along with a positive (histamine) and negative (saline) control (ALK Abelló). A mean wheal diameter of 3 mm or greater than elicited by the negative control was considered positive. The ovalbumin content of the AREPANRIX H1N1 AS03 Adjuvanted H1N1 vaccine used had a stated allowable limit of 165ng/ml.

## Results

**Figure 1:**
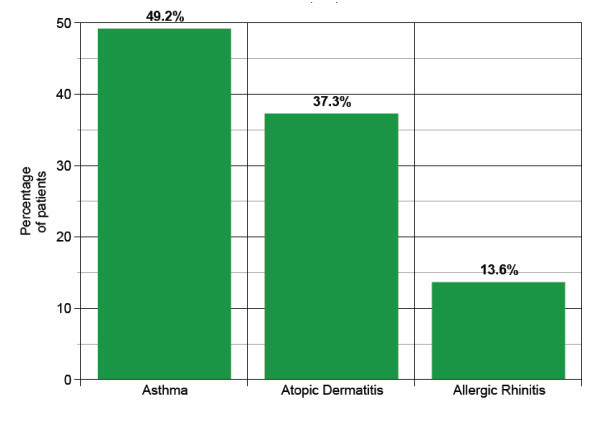
**Comorbidity of Asthma, Atopic Dermatitis and Allergic Rhinitis**.

There were a total of 61 patients with history of egg allergy referred to the pediatric and adult allergy and immunology clinics. 2 patients were excluded, one did not have skin prick testing to the vaccine (only vaccine administration), and the other passed an egg challenge during the study period. Of the remaining 59 patients, there were 41 (69.5%) males and 18 (30.5%) females ranging from age 1 through 27 years with a mean of 5.6 years. There were 8 control patients with no history of egg allergy, aged 13-77 years (mean 45.5 years) with 7 females (87.5%) and 1 male(12.5%). Demographics are shown in table 1. A comorbid diagnosis of asthma, or atopic dermatitis or allergic rhinitis was found in 29 (49.2%), 22 (37.3%), 8 (13.6%) respectively of all patients referred (Figure 1).

**Table 1:** Demographics and safety of H1N1 vaccine administration to study groups

	Patients with Egg allergy	Control Subjects
Number of patients	59	10

Age in years (mean)	1-27(5.6)	13-77(45.5)

Male sex (percentage)	41(69.5%)	1(12.5%)

Positive skin test response to H1N1 vaccine	1	0

Reaction post-vaccination	None	None

Exact 95% Confidence Interval for percentage who can safely receive this vaccine	90.23%, 99.67%	63.06%, 100%

**Figure 2:**
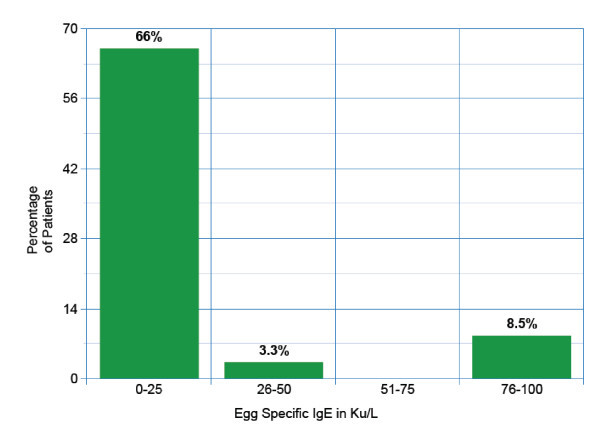
**Egg Specific IgE levels in Ku/L**.

**Figure 3:**
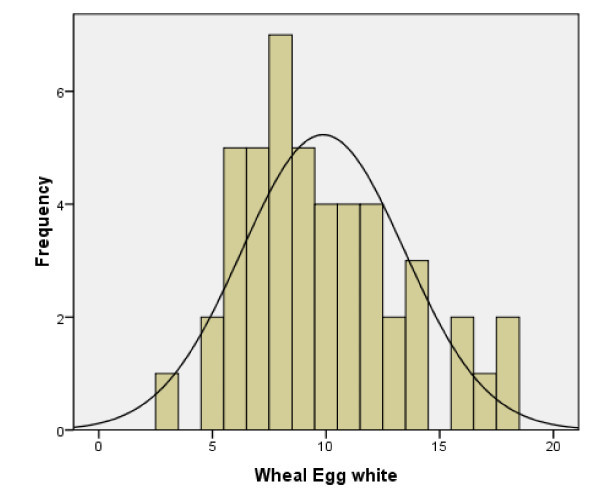
**Wheal to egg white in milimitres**.

All 59 patients had either a skin prick test performed to egg and/or a specific IgE level to egg white or egg yolk. 42 patients had both skin prick test to egg as well as specific IgE levels to egg performed. Of the 46 patients that had a specific IgE level performed to egg white, the mean egg white specific IgE level was 16.2Ku/L. Of the 47 patients that had skin prick testing to egg white commercial extract, the mean wheal size was 9.9 mm. Egg specific IgE levels and skin testing results are seen in Figure 2 and 3 respectively.

Skin testing to the H1N1 vaccine was performed in 59 patients and was found to be positive in only one (1.7%) patient. All patients with negative skin prick testing to the H1N1 vaccines were administered a dose of the H1N1 vaccine regardless of their risk category. The patients were then observed for 30 minutes after vaccination and no adverse events were noted. The one patient with a positive skin test had a H1N1 skin test diameter of 4 mm. Due to the H1N1 epidemic, it was decided that the benefits of vaccination outweighed the risks. After discussion with the patient's parents regarding the risks and benefits of vaccine administration or omission, the vaccine was administered to this individual, 10% of the dose initially then 90% with 30 minute observation after each injection. There were no adverse results.

Of note, this 2 year old male with a positive skin prick test to the H1N1 vaccine had a wheal diameter of 16 mm to egg white, comorbid diagnoses of asthma and atopic dermatitis as well as allergy to fish and shellfish. There were only 4 individuals with a larger or equivalent wheal diameter to egg than this patient with 2 at 18 mm and one at 17 mm and one at 16 mm. Of these four patients, one was not otherwise atopic, one had a comorbid diagnosis of asthma, two had comorbid diagnosis of both asthma and allergic rhinitis and none had allergy to fish or shellfish.

## Discussion

A novel 2009 influenza H1N1 virus was responsible for the first influenza pandemic in over 4 decades. H1N1 associated hospital admissions have been described in several Canadian studies [6,7]. Within months of the initial identified cases of H1N1, a vaccine was produced in hopes of reducing morbidity and mortality associated with this virus. Our series demonstrated that a negative skin prick test and challenge are highly predictive of safely tolerating vaccine administration. In the past, influenza vaccines have been contraindicated in patients with egg allergy due to the ovalbumin content of the vaccine. However, previous studies have demonstrated that patients with egg allergy can receive an influenza vaccine in a 2 dose protocol when the vaccine contains ≤ 1.2 micrograms/ml of egg protein after appropriate skin testing [8]. A recent Canadian study also suggested that skin testing to the flu vaccine in patients with egg allergy is not necessary. Here Gagnon et al found that none of the over 800 patients with confirmed egg allergy had symptoms of anaphylaxis in the follow up period [9].

Similarly, our series of 59 patients also demonstrates that a 1 dose protocol in children and adults can be safely tolerated when the vaccine contains ≤165 ng/mL of ovalbumin. In addition, an egg allergic patient with a minimally positive skin test of 4 mm also tolerated vaccine administration with no adverse events in two half hour observation periods.

## Conclusions

The H1N1 vaccine appears to be safe to administer to individuals with a diagnosis of egg allergy. One of our 59 (1.7%) patients did have a positive skin prick test to the H1N1 vaccine along with a positive skin prick test to egg white, elevated specific IgE levels to egg white and egg yolk, and tolerated vaccine administration via challenge. Administration of the H1N1 vaccine to individuals with egg allergy appears to be safe. This study has shown that almost all skin tests to the H1N1 vaccine were negative making the role of skin testing questionable. Administering the vaccine in a 1 or 2 dose protocol without skin testing is a reasonable alternative as per the CSACI guidelines.

## Competing interests

The authors declare that they have no competing interests.

## Authors' contributions

All authors read and approved the final manuscript.

## References

[B1] O'Riordan S Barton M Yau Y Read S Allen U Tran D Risk factors and outcomes among children admitted to hospital with pandemic H1N1 influenza CMAJ 2010 182 39 44 19926677 10.1503/cmaj.091724 PMC2802603

[B2] Tuite A Greer A Whelan M Winter A Lee B Yan P Wu J Moghadas S Buckeridge D Pourbohloul B Fisman D Estimated epidemiologic parameters and morbidity associated with pandemic H1N1 influenza CMAJ 2010 182 131 136 10.1503/cmaj.091807 19959592 PMC2817319

[B3] Zarychanski R Stuart T Kumar A Doucette S Elliot L Ketter J Plummer F Correlates of severe disease in patients with 2009 pandemic influenza (H1N1) virus infection CMAJ 2010 182 257 264 20093297 10.1503/cmaj.091884 PMC2826467

[B4] Waibel K Gomez R Ovalbumin content in 2009 to 2010 seasonal and H1N1 monovalent influenza vaccines J Allergy Clin Immunol 2010 125 749 751 10.1016/j.jaci.2009.12.015 20060576

[B5] The Canadian Society of Allergy and Clinical Immunology Statement: administration of H1N1 and seasonal influenza vaccine to egg allergic individuals http://www.csaci.ca/include/files/CSACI_H1N1_Statement.pdf Accessed July 23, 2010

[B6] Helferty M Vachon J Tarasuk J Rodin R Spika J Pelletier L Incidence of hospital admissions and severe outcomes during the first and second waves of pandemic (H1N1) 2009 CMAJ 2010 182 1981 7 10.1503/cmaj.100746 21059773 PMC3001504

[B7] Kumar A Zarychanski R Pinto R Critically ill patients with 2009 influenza infection in Canada JAMA 2009 302 1872 1879 10.1001/jama.2009.1496 19822627

[B8] James J Zeiger R Lester M Fassano M Gern J Mansfield L Schwartz H Sampson H Windom H Machtinger S Lensing S Safe administration of influenza vaccine to patients with egg allergy J Pediatrics 1998 133 624 8 10.1016/S0022-3476(98)70101-5 9821418

[B9] Gagnon R Primeau M Des Roches A Lemire C Kafan R Carr S Ouakki M Benoit M De Serres B Safe vaccination of patients with egg allergy with an adjuvanted pandemic H1N1 vaccine J Allergy Clin Immunol 2010 126 317 23 10.1016/j.jaci.2010.05.037 20579720

